# American Dental Association

**Published:** 1889-09

**Authors:** 


### EDITORIAL.

### American Dental Association.

The twenty-ninth annual meeting of the American Dental Association was held at Saratoga, August 6th to the 9th inclusive. The attendance was very good indeed, and the interest manifested in the work of the body was well sustained to the close. The scientific part of the proceedings were equal to that of former meetings, and in some respects perhaps better.

Some ethical questions of great importance were brought up for consideration, and received a goodly share of attention. Prominent among these was that of protection against the claims of those who make demands for money under patents (for instruments, appliances, materials or processes), the legal status of which has not been fully established.

The general feeling was, as shown in the discussion, that while the profession will respect and acquiesce in any and all claims having the unquestioned sanction of law, yet they will resist any and all demands that have not a good and sufficient foundation in law and right. The fact that some dentists yield to the terms of the International Tooth-crown Co., and are constantly paying that Co. money which is used in a war-fare on the dentists, was strongly condemned; it was maintained that the dentists should stand together, and utilize their resources in testing the validity of the patents under which that Co. is operating. As matters now are the profession is as a house divided against itself, and on that account labors under great disadvantage in this contest, while the Crown Co. is a unit, and operates through concentrated effort.

The subject of dental education came in for its share of consideration. There was a very hearty and unanimous concurrence in the action of the Association of Dental College Faculties, in regard to the extension of the time of college instruction for graduation to three terms instead of two. The utmost harmony and good feeling prevailed throughout the entire meeting.

Prof. M. W. Foster, of Baltimore, was elected President, and

Drs. Cushing and Fuller were re-elected Secretary and Treasurer. The next meeting will be held at Excelsior Springs, Mo.

The October No. of the *Register* will contain a full report of the proceedings.

